# Immunogenicity of adenovirus-vector vaccine targeting hepatitis B virus: non-clinical safety assessment in non-human primates

**DOI:** 10.1186/s12985-018-1026-3

**Published:** 2018-07-24

**Authors:** Xuefeng Zhang, Jing Wang, Jing Lu, Rongrong Li, Shuli Zhao

**Affiliations:** 1 Jiangsu Tripod Preclinical Research Laboratories Co., Ltd., Nanjing, 211800 China; 2 Nanjing First Hospital, Nanjing Medical University, 68 Changle Road, Nanjing, 210006 China

**Keywords:** Adenovirus vector, Gene therapy, Hepatitis B virus, Immunogenicity, Safety assessment

## Abstract

**Background:**

A new promising therapeutic approach has emerged for patients chronically infected by the hepatitis B virus (HBV) with the development of a non-replicative adenovirus vector vaccine candidate (Ad-HBV). The vaccine encodes a fusion protein composed of a truncated HBV core protein, mutated polymerase protein, and two envelope domains. In this study, we assessed the immunogenicity of Ad-HBV administered to cynomolgus monkeys during a non-clinical safety assessment.

**Methods:**

The virus was subcutaneously administered at 1.0 × 10^9^ viral particles (VP)/animal (low-dose group), 1.0 × 10^10^ VP/animal (mid-dose group), and 1.0 × 10^11^ VP/animal (high-dose group); the control groups were administered an Ad5-null virus (1.0 × 10^11^ VP/animal) and saline only.

**Results:**

Except for inflammatory cell infiltration under the skin at the injection sites and transient elevation of body temperature and serum albumin, no Ad-HBV-related toxic effects were noted in any treatment group. Moreover, interferon (IFN)-γ enzyme-linked immunospot assays showed that Ad-HBV induced the targeting of T cells to a broad spectrum of HBV-specific epitopes spanning all three of the selected HBV immunogens (core, polymerase, and envelope domains) in a dose-dependent manner. Although anti-Ad antibody was produced in all groups (except for the saline control), the antibody titers were significantly lower in the high-dose Ad-HBV group than in the group that received the same dose of the Ad-null empty vector. In addition, the IFN-γ and IL-2 expression levels in the liver were significantly improved for the mid-dose, high-dose, and Ad-null control group (*p* < 0.05), but not for the low-dose group.

**Conclusions:**

Taken together, this safety assessment indicates that the Ad-HBV candidate vaccine is a potent specific immunotherapeutic agent, supporting its further clinical development as an anti-HBV infection vaccine.

**Electronic supplementary material:**

The online version of this article (10.1186/s12985-018-1026-3) contains supplementary material, which is available to authorized users.

## Background

Hepatitis B virus (HBV) infection continues to be a serious clinical challenge, which induces the development of liver cirrhosis and hepatocellular carcinoma, even leading to mortality, in many areas of the world [1, 2]. Currently, the utility of first-line antiviral agents or immune system modulators recommended by the World Health Organization can only help to reduce and control the viral load and minimize liver damage, but cannot effectively clear the infection and seldom achieve a cure [3, 4]. Thus, these current therapies remain far from satisfactory. In particular, the wide spread of multidrug-resistant HBV strains is increasingly threatening the efficacy of currently available antiviral drugs [5, 6]. Therefore, there is an urgent need to actively develop and test promising novel therapies to target HBV.

Gene therapy is considered to be a newly effective option against many diseases by directly delivering therapeutic genes or those that can induce an improvement in the systemic immune response [7]. Owing to its efficient infectivity, high loading capacity, and stability outside of cells, adenovirus (Ad) is a promising candidate vector for gene delivery, and has been widely adopted for many gene therapy strategies developed to date [8]. Ad-HBV is considered to be the primary HBV immunotherapeutic developed through a transgenetic approach with the aim of application for treating patients chronically infected by HBV [9, 10]. Ad-HBV is a therapeutic vaccine candidate based on human E1- and E3-deleted replication-defective Ad serotype 5 (Ad5) vectors encoding an HBV fusion protein consisting of a truncated form of the HBV core domain (at the N-terminal) fused to a deleted and mutated HBV polymerase sequence, and two HBV envelope domains are inserted within the polymerase deletions. The nucleotide sequence encoding this HBV fusion protein is based on the HBV isolate Y07587, which was slightly modified [9]. This vaccine candidate was designed to stimulate specific T cell-based immunity similar to that seen in HBV resolvers.

Pharmacodynamics studies on Ad-HBV have been performed in three animal models thus far: HLA-A2 transgenic mice, C57BL6 mice, and BALB/c mice. In all cases, Ad-HBV was demonstrated to effectively induce T cells to produce various cytokines [interferon (IFN)-γ/tumor necrosis factor (TNF)-α and interleukin (IL)-2] in response to HBV antigen stimulation [9]. However, there were some potential disadvantages noted, including damage to the host immune response, hepatocytoxicity, and a short half-life; thus, non-clinical safety assessment is essential prior to further clinical development and trial as a vaccine. An unsolved challenge in the development of gene therapy drugs is that the vectors and their encoding proteins ultimately become immunogenic in some patients, thereby provoking an immune response resulting in immunotoxicity [11]. In contrast to therapeutic proteins whose immunogenicity is mainly evaluated by measuring the levels of anti-drug antibodies produced, there is currently no well-established and widely accepted method for the immunogenicity assessment of gene therapy drugs [12, 13]. Therefore, in the present study, we focused on evaluating the immunogenicity of the subcutaneous injection of Ads-HBV vaccines in a non-human primate at different doses to obtain reference data through a non-clinical safety assessment in preparation for a clinical trial.

## Methods

### Cell lines

The human lung cancer cell line A549 was purchased from Shanghai Cell Resource Center of the Chinese Academy and cultured in Dulbecco’s modified Eagle medium (DMEM)/F12 (Thermo Fisher Biochemical Products, Beijing, China) supplemented with antibiotics (100 U/mL penicillin and 0.1 mg/mL streptomycin; Invitrogen, Life Technologies) and 10% fetal bovine serum (Gibco, Life Technologies) at 37 °C in a 5% CO_2_ incubator.

### Ad-HBV production

All Ad vectors used in this study, including Ad5 not encoding any antigen (empty Ad, hereafter Ad-null), Ad5-luciferase (Ad5-Luc2), and Ad-HBV, were constructed, produced, purified, and titrated as described elsewhere [14–16]. Finally, the purified viruses were re-suspended in sterile phosphate-buffered saline (PBS) buffer and stored at − 80 °C. Virus quantification (number of virus particles, VP) was determined by high-pressure liquid chromatography.

### Non-clinical safety assessment in cynomolgus macaques

The long-term toxicity of Ad-HBV vaccines was evaluated in B virus- and Ad-free cynomolgus monkeys. *Macaca fascicularis* as the non-human primate model for the present safety assessment, which were obtained from Hainan New Source Biotech Co., Ltd. (Hainan, China). A total of 50 monkeys were used in this study, with equal numbers of males and females, weighing 2.5–4 kg (2.5–5-year-old). In brief, the monkeys were divided into five groups (*n* = 10 per group, five animals per sex) and injected with Ad-HBV at the following doses: 1.0 × 10^9^ VP/animal (low-dose group), 1.0 × 10^10^ VP/animal (mid-dose group), and 1.0 × 10^11^ VP/animal (high-dose group). Vehicle control (1 mL of 0.9% saline per animal) and injection of 1.0 × 10^11^ VP/animal Ad5-null were used as controls. All animals were injected subcutaneously weekly seven times in total, and the animals’ clinical symptoms were continually observed twice a day for 3 months. After gene therapy (3 days after the last dosing), six animals from each group (three animals per sex) were sacrificed for safety, toxicology, biodistribution, and other analyses. The remaining animals were left to recover for 4 weeks. Monitoring parameters were collected from each animal at several time points. The time schedule and experimental protocol are summarized in Table 1.

**Table 1:** Study design and methodology

Characteristic	Schedule
No. of animals	25 males/25 females
Groups	low-dose, mid-dose, high-dose, Ad5-null, and vehicle control group
Subcutaneous injection	Weekly × 7
Day killed	Days 46 and 71
Clinical signs	Twice daily
Body weight and temperature	Twice before injection, weekly after dosing
ECG	Pre-injection, day 44, and day 67
Clinical pathology (hematology, serum chemistry)	Pre-injection, day 21, day 44, and day 67
Serum IFN-γ	Pre-injection, day 7, day 35, day 45, and day 68
Lymphocyte subsets	Pre-injection, day 3, day 10, day 45, and day 68
Serum anti-Ad antibody, neutralizing antibody	Pre-injection, day 7, day 21, day 45, and day 68
Systemic autopsy, organ weight, histopathology	Day 46 and day 71
Hepatic IFN-γ, TNF-α, and IL-2	

### Flow cytometry analysis

To evaluate the immunoreaction of Ad-HBV, the lymphocyte subgroups in the peripheral blood were analyzed throughout the trial by a flow cytometry assay [17]. Peripheral blood lymphocytes from all animals were isolated using lymphocyte separation medium (TBDscience, Tianjin, China) density-gradient centrifugation according to the manufacturer’s instructions. The cells were then stained with specific conjugated antibodies (anti-CD4-PE, anti-CD8-APC, anti-CD45-PE Cy7, and anti-CD14-APC) and subjected to flow cytometry, respectively. The data were analyzed using FlowJo software.

### Immunohistochemistry (IHC) assay

IHC was used to determine the expression of IFN-γ, TNF-α, and IL-2 in the liver tissues collected from animals in each group. In brief, paraffin-embedded tissues were prepared from sacrificed animals, and 5-μm sections of liver tissues were prepared by extracting the paraffin in xylene. The sections were then re-hydrated, the endogenous peroxidase was inactivated by 3% H_2_O_2_ PBS, and then sections were treated with antigen repair solution (10 mM citrate solution; Maixin-Bio, Fujian, China) in a microwave oven at 95–100 °C for 15 min to retrieve the antigens. After blocking non-specific reactivity by 2% bovine serum albumin, the sections were incubated with antibodies against monkey IFN-γ (Ab25101, Abcam), TNF-α (Ab6671, Abcam), and IL-2 (500,302, Biolegend) for 2 h at 37 °C. After washing three times with PBS, the suitable secondary antibody labeled with horseradish peroxidase (HRP) was added to the sections and incubated for 1 h at 37 °C. The slides were then washed five times with PBS, and developed using a diaminobenzidine kit (Maixin-Bio, Fujian, China) according to the manufacturer’s instructions.

Immunoreactivity was graded into the following four groups based on the frequency of positive staining: − (negative), no specific staining or < 5% positive staining; +, ≥5 to < 30% positive staining; ++, ≥30 to < 70% positive staining; and +++, ≥70% positive staining. Grades − and + were considered to reflect low expression, and grades ++ and +++ were considered to reflect high expression [18].

### Enzyme-linked immunosorbent spot (ELISpot) assay of IFN-γ-secreting T cells

The quantity of IFN-γ-secreting T cells in the peripheral blood during this trial was assessed by ELISpot using the Monkey IFN-γ kit (3420 M-2HPW-2, MABTECH Inc., Cincinnati, OH, USA) following the manufacturer’s instructions [19, 20]. The peptides used in this study (Table 2) were previously identified as showing reactivity in a pilot experiment with cynomolgus monkeys, and were synthesized by Genscript Biological Technology Co., Ltd. (Nanjing, China). For each library, peptides were dissolved in 100% dimethyl sulfoxide (DMSO, Sigma) and pooled at a concentration of 2 mg/mL per peptide, and each pool was tested at a final concentration of 5 μg/mL per peptide in the ELISpot assays. Three peptide pools were used as stimulatory agents. Hence, the ELISpot assays were carried out in five groups, including the negative control (unrelated peptide in DMSO, DLMDLMGYIPLV), core pool, Env pool, Pol pool, and positive control (anti-CD3 IgG, 1:1000, provided in the kit).

**Table 2:** Peptides used for ELISpot tests

Group	Peptide Sequences
Core	H-QAILCWGELMTLATW-OH H-CWGELMTLATWVGGN-OH H-LMTLATWVGGNLEDP-OH H-ATWVGGNLEDPISRD-OH H-GGNLEDPISRDLVVS-OH H-EDPISRDLVVSYVNT-OH H-SRDLVVSYVNTNMGL-OH H-VVSYVNTNMGLKFRQ-OH
Env	H-AFGKFLWEWASARFS-OH H-FLWEWASARFSWLSL-OH H-WASARFSWLSLLVPF-OH H-RFSWLSLLVPFVQWF-OH H-LSLLVPFVQWFVGLS-OH H-VPFVQWFVGLSPTVW-OH H-QWFVGLSPTVWLSVI-OH H-GLSPTVWLSVIWMMW-OH
Pol8	H-RRSFGVEPSGSGHST-OH H-GVEPSGSGHSTNLAS-OH H-SGSGHSTNLASKSAS-OH H-HSTNLASKSASCLYQ-OH H-LASKSASCLYQSPVR-OH H-SASCLYQSPVRKAAY-OH H-LYQSPVRKAAYPAVS-OH H-PVRKAAYPAVSTFEK-OH
Pol11	H-DWGPCAEHGEHHIRI-OH H-CAEHGEHHIRIPRTP-OH H-GEHHIRIPRTPARVT-OH H-IRIPRTPARVTGGVF-OH H-RTPARVTGGVFLVDK-OH H-RVTGGVFLVDKNPHN-OH H-GVFLVDKNPHNTAES-OH H-VDKNPHNTAESRLVV-OH H-PHNTAESRLVVDFSQ-OH
Pol13	H-TNLLSSNLSWLSLDV-OH H-SSNLSWLSLDVSAAF-OH H-SWLSLDVSAAFYHLP-OH H-LDVSAAFYHLPLHPA-OH H-AAFYHLPLHPAAMPH-OH H-HLPLHPAAMPHLLPL-OH H-HPAAMPHLLVGSSGL-OH H-MPHLLVGSSGLSRYV-OH
Negative control	DLMDLMGYIPLV
Positive control	Anti-CD3 IgG

In brief, approximately 5 mL of peripheral blood was collected to isolate the lymphocytes from each sample in tubes with heparin. Lymphocytes (2 × 10^5^/well) in triplicates were plated and incubated with complete DMEM containing different peptides or control agents for 36 h in a 37 °C humidified incubator with 5% CO_2_. The medium was then removed and the plates were washed five times with PBS. HRP-conjugated anti-IFNγ (100 μL, 1:200) was added to each well, and the plates were washed five times with PBS after incubation for 2 h at room temperature. Finally, TMB substrate solution was used to detect the IFNγ-positive spots. After the plate dried, the spots in each well were inspected and counted under a dissection microscope.

### Detection of anti-ad antibodies and its neutralization activity

The anti-Ad-HBV antibody titers were measured by indirect enzyme-linked immunosorbent assay (iELISA) as previously described [21]. In brief, binding of the anti-drug antibodies in serum samples with pre-coated inactivated antigen on a microtiter plate (1.0 × 10^8^ VP/100 μL carbonate/bicarbonate buffer per well) was detected with HRP-labeled anti-monkey immunoglobulin G (IgG). Based on a cut-off point of optical density values at 450 nm, the positive and negative pre-administration serum samples from the same animals were screened out and their neutralizing activity was further measured using an adapted luciferase-based virus neutralization assay [22]. In brief, the diluted serum samples (1/100, 1/500, and 1/1000) were mixed with 1.0 × 10^6^ VP of Ad5-Luc2 in complete DMEM, which is a luciferase-expressing Ad vector (ShenZhao Biotechnology, China), and incubated for 1 h at room temperature. Then, 5 × 10^4^ A549 cells were added to the medium and incubated at 37 °C, 5% CO_2_ for 24 h. The luciferase assay system (Promega) was used to measure luciferase activity in the cells with a microplate reader (BioTek). Finally, neutralizing antibody activity in the blood of each animal after administration was evaluated according to the following formula. Neutralization efficiency (%) = [(Mean fluorescence intensity of the pre-administration control group − mean fluorescence intensity of the administration group)/mean fluorescence intensity of pre-administration control group] × 100%.

### Statistical analysis

Data are presented as mean ± standard deviation of each group, which were analyzed using the SPSS 10.0 software packet. Comparisons between groups were tested by one-way ANOVA, measurement data was analyzed by the chi-square test, and rank data were analyzed by Fisher’s exact probability. Data of dose groups were compared with those of the vehicle control group; *p* < 0.05 was deemed to reflect a statistically significant difference.

## Results

### Non-clinical safety assessment

**Fig. 1:**
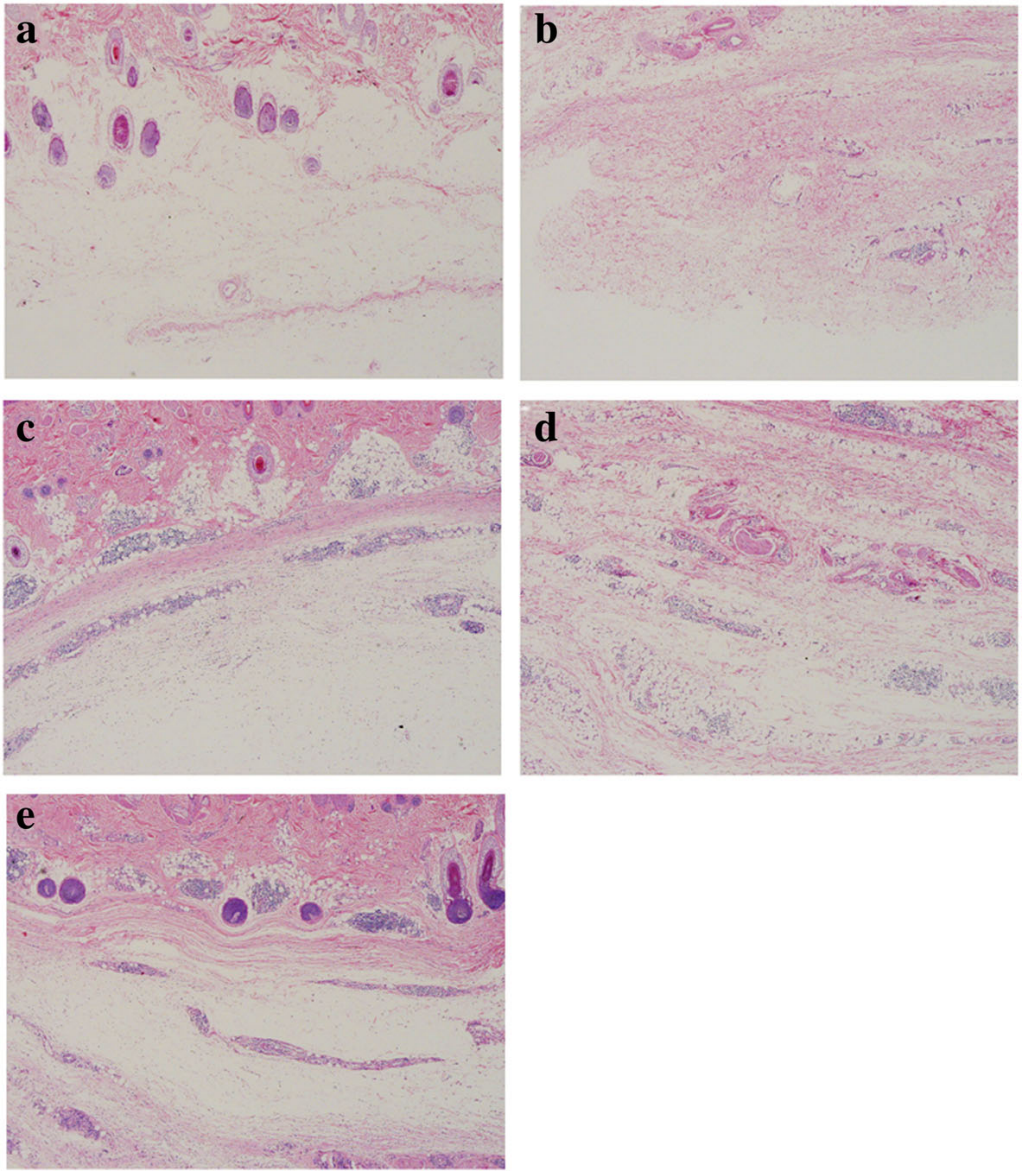
Representative histological sections stained with hematoxylin and eosin (HE) in the skin tissues at the injection site. Vehicle control group (**a**), low-dose group (**b**), mid-dose group (**c**), high-dose group (**d**), and Ad5-null control group (**e**). Infiltration of inflammatory cells (plasma cells, lymphocytes) was detected in the injected skin tissues (**b**–**e**). Images were captured under an Olympus BX50 microscope (magnification × 40)

Erythema was observed at the injection site of the skin at different proportions in the animals of the Ad-HBV groups (low-dose, 6/10; mid-dose, 6/10; high-dose, 4/10) and Ad5-null group (5/10), but was not detected in the vehicle control group. Histological examination showed infiltration of inflammatory cells (plasma cells, lymphocytes) under the skin at the injection site, representing a local immune response caused by administration of the Ad vector (Fig. 1). Compared with the vehicle control group, transient elevation of ALB (day 21, 44, and 67) and TP (day 67) levels was detected along with an increase in body temperature (day 5, 12, 19, 26, 33, and 40) in the Ad-HBV and Ad-null groups after injection. However, these levels did not change substantially before and after vector administration and fluctuated within the normal physiological range (Additional file 1: Table S1, Additional file 2: Table S2 and Additional file 3: Table S3). Thus, these effects did not reflect a significant toxicological reaction.

Compared to the vehicle control group, there was a significant difference in lymphocyte subsets detected for CD8+ T cells in the low-dose group and macrophages (CD45 + CD14+) in the Ad-null group; however, these changes were noted for a single index and were not dose-dependent, indicating that they were of minimal biological significance. The other parameters fluctuated within normal ranges throughout the study (Additional file 4: Table S4).

In addition, throughout the course of the study, no significant differences were observed in body weight, food consumption, ECG, and other clinical pathological parameters such as systemic autopsy, organ weight, and histopathology (data not shown). Overall, these findings demonstrated that the main toxicity of Ad-HBV is related to stimulation at the injection site by the Ad itself.

### T cell-specific secretion of IFN-γ induced by HBV antigen

To confirm the potent and functional cellular immune response in non-human primates resulting from Ad-HBV injection during this non-clinical safety assessment, we monitored the HBV-specific immune response using IFN-γ ELISpot assays.

**Fig. 2:**
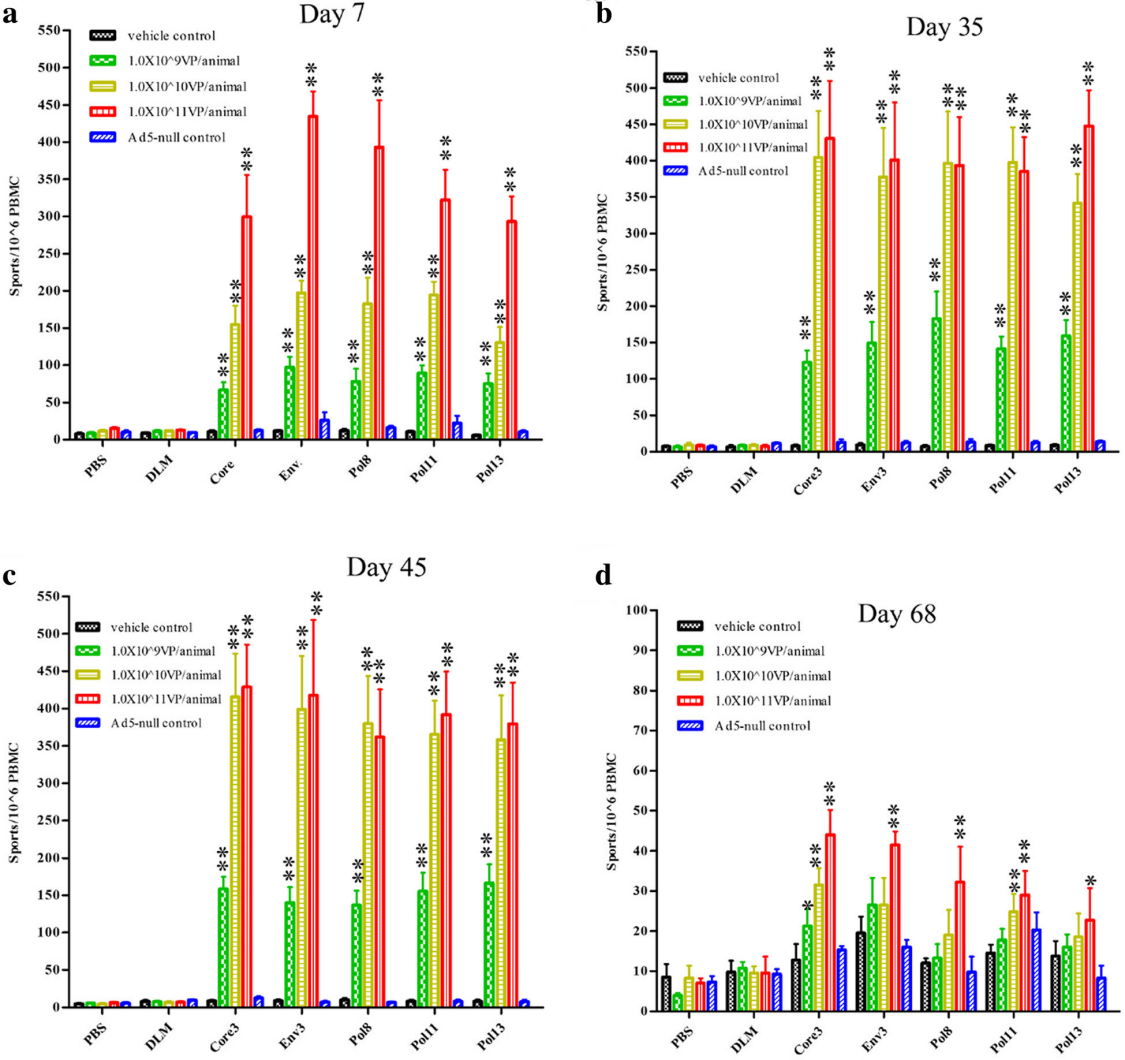
Long-term monitoring of HBV-specific cells following multiple administrations (day 1, 8, 15, 22, 29, 36, and 43) up to 68 days post-injection. Cynomolgus monkeys were injected subcutaneously with a low dose (1.0 × 10^9^ VP/animal), mid dose (1.0 × 10^10^ VP/animal), and high dose (1.0 × 10^11^ VP/animal) of Ad-HBV, or with an empty Ad-null vector (1.0 × 10^11^ VP/ animal); an equal volume of saline was injected as a negative control. HBV antigen-specific cells were monitored and quantified by interferon-γ (IFNγ) ELISpot assays conducted at day 7(**a**), 35(**b**), 45(**c**), and 68(**d**) post-injection using peripheral blood mononuclear cells (PBMCs) respectively stimulated by HBV peptide pools (Core, Env, pol8, pol11, and pol13) or irrelevant peptides (DLM). *p < 0.05, **p < 0.01 vs. vehicle control

Although the numbers of HBV antigen (Core, Env, and Pol)-specific IFNγ+ cells in the peripheral blood mononuclear cells (PBMCs) were significantly higher for all three dose groups (low, mid, and high) compared to those in the vehicle control group and Ad5-null control group (*p* < 0.01) at day 7, 35, and 45, the cell numbers in the low-dose group (10^9^ VP/animal) were significantly lower (*p* < 0.01) than those in the mid-dose (10^10^ VP/animal) and high-dose (10^11^ VP/animal) groups (Fig. 2). In addition, although the the numbers of HBV antigen-specific IFNγ+ cells in PBMCs for the mid-dose group were significantly lower than those of the high-dose group at the first administration on day 7 (*p* < 0.05), there was no difference between these groups at days 35 and 45. These results indicated that the dose setting in this toxicity assessment test was reasonable, and 10^10^ VP/animal was the highest effective dose in the Ad-HBV test of cynomolgus macaques.

### Anti-drug antibodies and neutralization activity

**Fig. 3:**
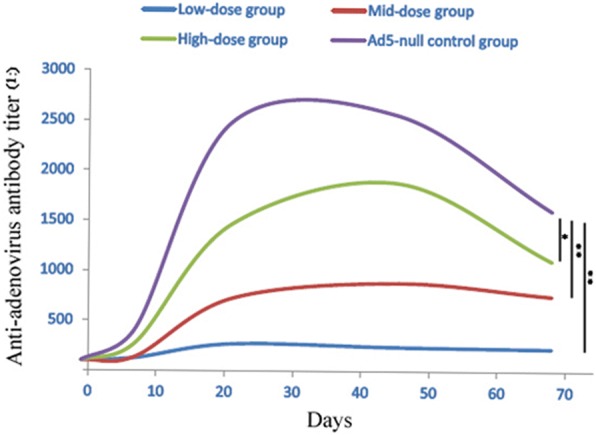
Serum antibody titer levels following multiple administrations (day 1, 8, 15, 22, 29, 36, and 43) up to day 68 post-injection. The cut-off titer value was 1:100. Antibody titers of each group are respectively shown as purple (Ad5-null control), green (high-dose group), red (mid-dose group), and blue (low-dose group) curves. Antibody titers were compared using a non-parametric test (Friedman’s test with Dunn’s multiple comparisons test); ** *p* value < 0.01

The binding and neutralization activity of anti-drug antibodies to the Ad vector-based vaccines may affect the clinical efficacy, alter the pharmacokinetic profiles, or cause adverse effects. Therefore, we used the anti-Ad antibody-negative animals in the non-clinical safety assessment test and monitored the levels of anti-Ad antibody in the serum after Ad-HBV administration. The levels of anti-Ad antibodies dose-dependently increased after the administration of Ad-HBV, and reached a peak value at day 21 that was sustained until the last administration (day 45). The levels of anti-Ad antibodies in the Ad5-null control group (Ad empty vector, 10^11^ VP/animal) were higher than those detected for the animals that received the same-dose (high-dose 10^11^ VP/animal) of Ad-HBV group, which may have resulted from gene inserting of truncated Pol, Core and Env antigen. In addition, the antibody titers were sustained at higher levels after 4 weeks of recovery (day 68) (Fig. 3).

The neutralization activity of the highest-titers serum samples from every animal in the Ad5-null control group and the three Ad-HBV groups (low, mid, and high doses) were then measured by the Ad vector luciferase-expressing inhibition assay. At 24 h of infection, the fluorescence intensity in the A549 cells gradient increased in proportion with the Ad5-Luc2 concentration. To improve the sensitivity and sensitivity of this model, we selected 10^6^ VP/mL of Ad5-Luc2 to infect A549 cells in the neutralization experiment.

**Fig. 4:**
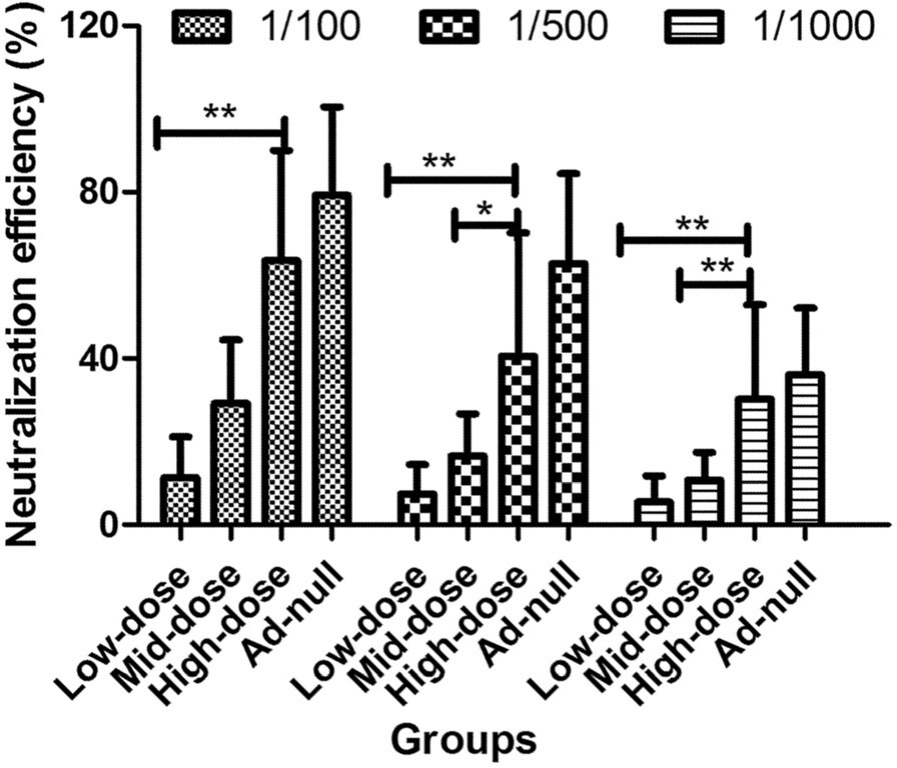
Neutralization of A549 cells with a luciferase-expressing adenovirus (Ad5-Luc2). Neutralization was determined by transgene expression inhibition in cells pretreated with mixed serum at different dilutions: 1:100, 1:500, and 1:1000. Neutralization efficiency was calculated from triplicate measurements; quadrate measurements are shown for 1.0 × 10^6^ viral particles (VP) of Ad5-Luc2 and 5 × 10^4^ A549 cells per well. *p < 0.05 and **p < 0.01

Compared with the pre-dosing serum samples, diluted serum samples (1/100, 1/500, and 1/1000) from all anti-drug antibody-positive animals decreased the fluorescence intensity to different extents in the infected A549 cells by Ad5-Luc2. The serum neutralizing effects of the high-dose and Ad-null groups were significantly higher than those of the low-dose and mid-dose groups, indicating that the anti-drug antibody-positive serum inhibited the infection activity of Ad5-Luc2 and that the neutralization activity is dose-dependent (Fig. 4). Thus, although the anti-Ad antibody was produced in almost all animals after the first immunization, these antibodies could partially neutralize the ability of Ad infection.

### Histology

**Fig. 5:**
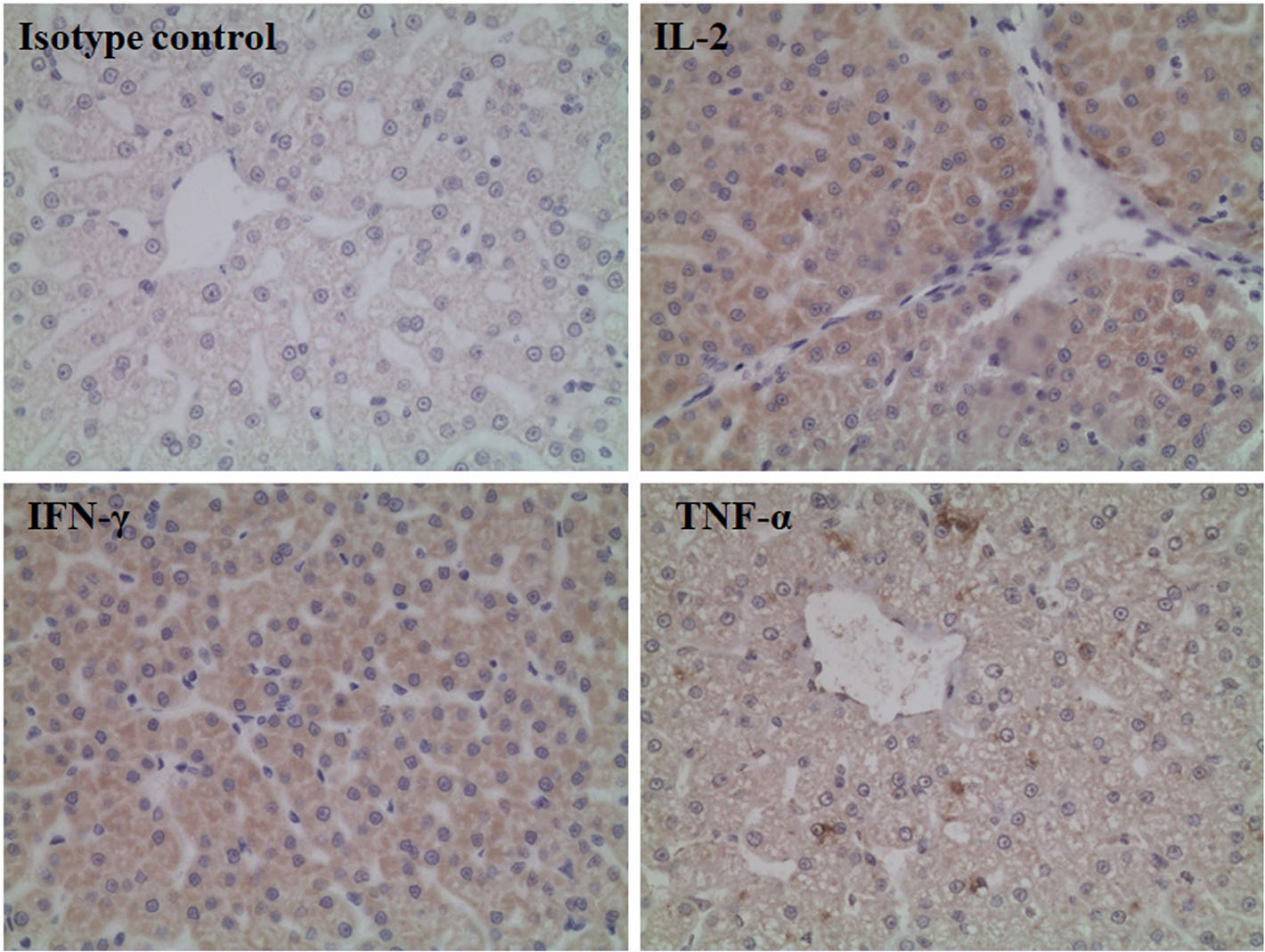
Representative images of positive immunohistochemical staining for IL-2, TNF-α, and IFN-γ in cynomolgus monkey liver tissues. Images were taken using the Olympus BX50 microscope (magnification, × 100)

Since Ad vectors are predominantly sequestered by the liver after administration, and a previous study revealed that Ad infection could significantly improve the expression of antiviral immune cytokines, we analyzed the levels of IFN-γ, IL-2, and TNF-α proteins in the liver by IHC to explore the immune response strategy in the liver. As shown in Table 3 and Fig. 5, administration with Ad-HBV significantly improved the IFN-γ and IL-2 expression levels of the livers in the mid-dose, high-dose, and Ad-null control group (*p* < 0.05), but not in the low-dose group. This suggested that the increased expression of these two cytokines in the liver should be related to the amount of Ad vector injected. However, there was no correlation between the TNF-α expression and administration of Ad-HBV.

**Table 3:** TNF-α, IFN-γ, and IL-2 protein expression in the liver

	Staining	Groups				
		Vehicle control	Low-dose	Mid-dose	High-dose	Ad-null
TNF-α	Low (%)	4 (40%)	3(30%)	2 (20%)	4 (40%)	5 (50%)
	High (%)	6 (60%)	7(70%)	8 (80%)	6 (60%)	5 (50%)
IFN-γ	Low (%)	10 (100%)	10 (100%)	5 (50%)	4 (40%)	5 (50%)
	High (%)	0(0%)	0 (0%)	5 (50%)^*#^	6 (60%)^*#^	5 (50%)^*#^
IL-2	Low (%)	8(80%)	7 (70%)	1 (10%)	3 (30%)	3 (30%)
	High (%)	2(20%)	3 (30%)	9 (90%)^*#^	7 (70%)^*#^	7 (70%)^*#^

^*^and ^#^*p* < 0.05 compared with the vehicle control or low-dose group, respectively

## Discussion

Several studies have been conducted on the application of gene therapies using Ad5-based vaccines for the treatment of cancer or chronic virus infections, which have shown that the immunogenicity responses specific for Ad proteins and for transgene-encoded proteins induced by vaccines could have a significant impact on the efficacy of a recombinant Ad vaccine [23–25]. Thus, an essential aspect to ensure the efficacy of virus-vector drugs is to comprehensively and accurately evaluate their immunogenicity. Here, we evaluated the basic immunogenic characteristics of an Ad-HBV vaccine by monitoring the induced HBV-specific T cell responses in a GLP three-month repeat dose toxicity study using cynomolgus monkeys as a non-human primate model.

In general, as a new and extensively- accepted therapy option, the major toxicological effects observed possibly associated with the activation of the immune response induced by Ad-HBV, for example erythema on the injection point skin and abnormalities of peripheral blood lymphocyte subsets. Although there were transient increase in the other physiological parameters (WBC, CREA, TP, ALB, et al.) compared with vehicle control group, their values were still within normal ranges, and so they did not have toxicological significance.

Compared with vehicle control group, there were some significance (p<0.05 or p<0.01) in some parameters (CD3 + CD8 + T cell, CD4+/CD8+, CD45 + CD14+) in low-dose, mid-dose and Ad5-null groups in different days, but it mostly was change in a single index and no dosing depended, so it was no biological significance.

Although the anti-adenovirus antibody were produced in almost all animals after first immunization and these antibody could partially neutralize the ability of adenovirus infection, Ad-HBV still induced strong and broad IFNγ+ cell responses and displayed a whole array of immunogenic characteristics, which showed the highly immunogenic potential of Ad-HBV vaccine. Because adenovirus vectors are predominantly sequestered by the liver after administration, we analyzed the levels of IFN-γ and IL-2 protein in the live by immunochemistry assay, and found that these two antiviral cytokines in the high-dose and Ad5-null control group were higher expressed than those in the vehicle control group.

The current study has several limitations. First, Natural killer (NK) cells naturally enriched in liver [26], and both of HBV infection [27] and adenovirus vector delivery [28] can affect the function of NK cells. However, we did not properly evaluate the function of NK cells in this study. In addition, it should be noted, we did not further investigate which cells targeted the 3 HBV antigens/domains encoded by Ad-HBV and produced the antiviral cytokines (IFN-γ and IL-2).

Overall, this non-clinical safety assessment showed that the Ad-HBV immunotherapeutic product induced no biologically significant toxicity, induced strong and broad IFNγ+ cell responses, and displayed a whole array of immunogenic characteristics, demonstrating the high immunogenic potential of the Ad-HBV vaccine. Moreover, this candidate vaccine has the advantage of gathering three HBV antigens in one vector together with the capacity to induce potent antiviral responses and broad immunogenic characteristics. Thus, Ad-HBV should be selected as a lead candidate for clinical development.

## Additional files

Additional file 1: **Table S1.** Effects of Ad-HBV administration on Hematology. (PDF 198 kb)

Additional file 2: **Table S2.** Effects of Ad-HBV administration on serum chemistry. (PDF 208 kb)

Additional file 3: **Table S3.** Effects of Ad-HBV administration on body temperature. (PDF 173 kb)

Additional file 4 **Table S4.** Effects of Ad-HBV on Lymphocytes subsets. (DOCX 55 kb)

## Abbreviations

Ad: adenovirus
DMEM: Dulbecco’s modified Eagle medium
DMSO: dimethyl sulfoxide
ELISpot: enzyme-linked immunosorbent spot
HBV: hepatitis B virus
HRP: horseradish peroxidase
IFN: interferon
IgG: immunoglobulin G
IHC: immunohistochemistry
IL: interleukin
PBMC: peripheral blood mononuclear cell
Pol: polymerase
TNF: tumor necrosis factor
VP: viral particle
