# Correction to: Immunogenicity of adenovirus-vector vaccine targeting hepatitis B virus: non-clinical safety assessment in non-human primates

**DOI:** 10.1186/s12985-018-1049-9

**Published:** 2018-09-03

**Authors:** Xuefeng Zhang, Jing Wang, Jing Lu, Rongrong Li, Shuli Zhao

**Affiliations:** 1 Jiangsu Tripod Preclinical Research Laboratories Co., Ltd., Nanjing, 211800 China; 2 Nanjing First Hospital, Nanjing Medical University, 68 Changle Road, Nanjing, 210006 China

## Correction

Ad-HBV is a product (T101) aiming to treat chronic HBV firstly developed by Transgene S.A., France then by Transgene Tasly (Tianjin) Biopharmaceutical Co., Ltd., Tianjin, China in China. So, in the originally published article [1], the “Ad-HBV” appeared throughout the whole paper for 41 times should be replaced by “T101”.

Additionally, in the “Acknowledgements” section in the originally published article, “The Ad-HBV were a kind gift from Xia Meng (Transgene Tasly (Tianjin) Biopharmaceutical Co., Ltd., Tianjin, China). We want to thank Zhiming Wang and Xiaomin Wang (Transgene Tasly (Tianjin) Biopharmaceutical Co., Ltd., Tianjin, China) for teaching neutralization activity detection of anti-Ad antibodies” should be replaced by “We want to thank Transgene Tasly (Tianjin) Biopharmaceutical Co., Ltd., to provide us the opportunity to participate in the non-clinical safety assessment of T101, and most of the research work appeared in this paper came from the non-clinical safety assessment of T101”.
