# Supplementary material for: Immunogenicity of adenovirus-vector vaccine targeting hepatitis B virus: non-clinical safety assessment in non-human primates
Source: Virol J. 2018 Jul 24;15:111. doi: 10.1186/s12985-018-1026-3 (PMC6056916; doi:10.1186/s12985-018-1026-3)
Supplement: Supplementary file 1 — Table S1. Effects of Ad-HBV administration on Hematology. (PDF 198 kb) [file 12985_2018_1026_MOESM1_ESM.pdf]

Additional file 1: Table S1 Effects of Ad-HBV administration on Hematology

Parameter	Time (d)	Vehicle	Low-dose 1.0×10^9 VP/animal	Mid-dose 1.0×10^10 VP/animal	High-dose 1.0×10^11 VP/animal	Ad5-null control 1.0×10^11 VP/animal
WBC	d-5	14.08±4.18	12.76±3.33	10.78±3.22	12.17±2.71	13.60±2.30
	d21	10.46±2.53	8.81±2.60	8.56±2.61	10.37±3.14	8.16±1.80
	d44	12.97±2.66	11.67±3.60	10.37±2.75	9.55±2.83*	9.55±2.37*
	d67	16.39±2.49	12.93±4.86	10.91±3.01	10.18±2.72	11.39±1.54
RBC	d-5	5.25±0.53	5.39±0.56	5.30±0.46	5.31±0.50	5.29±0.27
	d21	5.49±0.27	5.39±0.39	5.41±0.29	5.44±0.35	5.39±0.26
	d44	5.83±0.33	5.72±0.35	5.58±0.37	5.62±0.31	5.58±0.27
	d67	5.71±0.22	5.52±0.19	5.61±0.52	5.71±0.60	5.62±0.49
HGB	d-5	125±12	128±12	127±12	126±12	126±8
	d21	133±10	131±8	132±7	131±8	130±5
	d44	141±8	138±6	136±11	134±7	135±6
	d67	134±4	132±2	133±15	132±12	133±13
HCT	d-5	39.7±3.9	40.7±3.7	40.3±3.6	40.1±3.4	40.2±2.5
	d21	42.0±3.1	40.3±2.4	40.5±2.0	40.6±2.8	40.5±1.6
	d44	43.6±2.6	42.4±2.0	41.7±2.7	41.1±2.2	41.9±1.7
	d67	45.5±3.5	43.7±1.4	44.0±5.5	44.4±3.3	44.5±3.6
MCV	d-5	75.7±4.1	75.7±3.7	76.0±2.6	75.7±3.0	75.9±2.5
	d21	76.5±4.4	74.8±2.5	75.0±3.3	74.7±3.5	75.3±2.5
	d44	74.9±3.6	74.3±2.4	74.8±3.3	73.3±3.1	75.3±1.9
	d67	79.5±4.4	79.2±0.4	78.2±4.1	78.1±3.9	79.4±3.1
MCH	d-5	23.8±1.3	23.9±1.1	24.0±1.0	23.7±1.2	23.8±0.7
	d21	24.3±1.3	24.3±1.0	24.4±1.0	24.1±1.2	24.1±0.7
	d44	24.3±1.4	24.2±1.0	24.3±1.0	23.9±1.2	24.2±0.8
	d67	23.4±0.7	23.9±0.6	23.7±0.5	23.3±1.8	23.7±1.2
MCHC	d-5	314±8	316±10	315±11	313±14	313±7
	d21	318±10	325±10	325±13	322±15	320±9
	d44	324±10	326±9	325±14	326±12	321±7
	d67	295±16	302±7	304±14	298±14	299±11
RDW	d-5	14.1±1.6	13.5±0.8	13.7±0.9	14.2±0.7	14.0±0.9
	d21	13.4±0.8	12.8±0.8	13.2±0.7	13.2±0.7	13.3±0.8
	d44	12.2±0.4	11.8±0.7	12.0±0.5	12.1±0.6	12.3±0.5
	d67	12.2±0.2	11.8±0.5	11.9±0.5	11.9±0.4	12.1±0.4
PLT	d-5	416±72	417±116	356±67	384±100	359±73
	d21	483±72	462±129	461±105	434±98	424±95
	d44	428±81	405±93	400±94	371±78	359±76
	d67	456±71	434±108	368±122	332±82	362±79
MPV	d-5	9.6±0.8	9.1±1.0	9.8±1.3	9.4±0.9	10.0±1.0
	d21	9.1±0.9	8.6±0.7	9.4±1.1	9.3±0.7	9.8±1.2
	d44	9.3±0.9	9.0±0.7	9.5±1.1	9.0±0.5	9.8±1.4

Parameter	Time (d)	Vehicle	Low-dose 1.0×10^9 VP/animal	Mid-dose 1.0×10^10 VP/animal	High-dose 1.0×10^11 VP/animal	Ad5-null control 1.0×10^11 VP/animal
	d67	9.4±0.9	9.0±0.8	10.1±1.9	9.5±1.0	10.0±2.0
PDW	d-5	46.6±3.3	45.3±5.6	47.4±6.3	48.0±3.9	50.6±5.5
	d21	47.8±4.3	46.2±3.6	47.1±6.6	45.5±2.3	51.1±4.8
	d44	47.8±5.2	47.2±4.3	48.8±7.3	48.2±3.1	53.8±5.2
	d67	48.7±2.7	47.6±5.9	52.7±9.2	49.1±3.9	52.6±4.9
PCT	d-5	0.40±0.05	0.38±0.10	0.34±0.06	0.36±0.08	0.36±0.06
	d21	0.43±0.05	0.39±0.10	0.43±0.08	0.40±0.09	0.41±0.08
	d44	0.39±0.05	0.36±0.07	0.37±0.06	0.33±0.06	0.35±0.06
	d67	0.42±0.04	0.39±0.10	0.35±0.05	0.31±0.05	0.35±0.05
%NEUT	d-5	48.9±9.8	44.7±13.6	43.2±12.8	48.5±16.0	50.4±9.9
	d21	36.3±10.4	27.6±8.0	31.9±10.1	45.0±15.6	35.1±6.8
	d44	34.2±5.6	33.0±9.8	37.4±13.8	36.5±14.4	39.8±10.4
	d67	43.2±10.1	30.8±10.6	31.3±9.7	33.3±14.3	38.9±10.3
%LYMPH	d-5	45.7±9.4	49.9±13.2	45.3±12.5	45.4±15.4	44.8±9.3
	d21	56.8±9.8	65.5±8.7	60.5±8.6	48.9±14.5	59.2±6.5
	d44	58.6±5.0	61.1±10.0	55.5±12.5	55.3±13.4	54.9±9.5
	d67	50.2±9.0	64.9±10.6	61.9±8.7	61.4±14.5	56.5±10.2
%MONO	d-5	3.1±0.5	3.1±0.5	9.6±16.1	3.9±1.5	3.0±0.8
	d21	4.0±1.3	3.3±0.9	4.6±1.3	3.7±1.5	3.2±1.0
	d44	3.8±0.9	3.2±0.7	4.9±1.3	5.7±2.3	3.7±1.1
	d67	3.5±0.2	2.6±0.3	4.4±1.0	3.3±1.3	3.2±1.4
%EOS	d-5	1.5±1.7	1.7±1.6	1.3±1.1	1.5±1.8	1.2±0.7
	d21	2.1±1.6	2.7±2.0	2.3±2.4	1.7±1.2	1.6±1.2
	d44	2.6±2.7	1.9±1.3	1.6±1.7	1.8±2.0	0.9±0.5
	d67	2.4±2.1	1.0±0.6	1.8±1.2	1.5±0.9	0.8±0.7
%BASO	d-5	0.2±0.1	0.2±0.0	0.2±0.1	0.2±0.1	0.2±0.1
	d21	0.2±0.1	0.3±0.1	0.2±0.1	0.2±0.1	0.3±0.1
	d44	0.2±0.1	0.2±0.1	0.2±0.1	0.2±0.1	0.2±0.1
	d67	0.3±0.1	0.3±0.1	0.3±0.1	0.2±0.1	0.2±0.1
%LUC	d-5	0.6±0.2	0.5±0.2	0.4±0.1	0.5±0.2	0.5±0.2
	d21	0.6±0.2	0.6±0.1	0.5±0.2	0.5±0.2	0.7±0.2
	d44	0.6±0.2	0.5±0.1	0.4±0.2	0.5±0.2	0.5±0.2
	d67	0.6±0.2	0.5±0.1	0.4±0.2	0.4±0.1	0.5±0.2
%RETIC	d-5	2.32±1.43	2.07±0.47	2.09±0.64	2.24±0.71	2.01±0.46
	d21	1.77±0.52	1.71±0.56	1.77±0.52	1.48±0.42	1.68±0.22
	d44	1.02±0.33	1.14±0.31	1.22±0.42	1.03±0.36	1.19±0.37
	d67	1.22±0.11	0.90±0.16	1.13±0.11	0.82±0.16	1.14±0.42

[a]Data expressed as mean±SD.

*$p < 0.05$ compared with the vehicle control.
