# Supplementary material for: Immunogenicity of adenovirus-vector vaccine targeting hepatitis B virus: non-clinical safety assessment in non-human primates
Source: Virol J. 2018 Jul 24;15:111. doi: 10.1186/s12985-018-1026-3 (PMC6056916; doi:10.1186/s12985-018-1026-3)
Supplement: Supplementary file 2 — Table S2. Effects of Ad-HBV administration on serum chemistry. (PDF 208 kb) [file 12985_2018_1026_MOESM2_ESM.pdf]

Additional file 2: Table S2 Effects of Ad-HBV administration on serum chemistry

Parameter	Time (d)	Vehicle	Low-dose 1.0×10^9 VP/animal	Mid-dose 1.0×10^10 VP/animal	High-dose 1.0×10^11 VP/animal	Ad5-null control 1.0×10^11 VP/animal
ALT	d-5	92.6±57.6	77.3±39.7	82.9±61.3	72.7±23.8	90.6±47.5
	d21	54.1±13.2	59.1±31.5	54.4±17.8	76.5±47.1	87.2±50.0
	d44	70.1±28.6	77.9±42.0	60.4±21.3	82.1±58.6	98.2±53.8
	d67	57.8±11.1	54.0±23.3	77.3±69.5	84.5±65.8	162.5±140.5
AST	d-5	63.2±21.0	66.8±24.8	60.3±15.7	60.5±10.0	54.8±11.7
	d21	51.1±7.8	63.1±18.7	52.6±13.9	53.9±13.9	49.1±11.1
	d44	44.3±10.4	53.0±17.5	43.7±7.6	45.1±13.4	39.8±7.6
	d67	37.7±2.4	47.4±15.5	33.4±11.5	45.0±13.3	43.0±14.6
GGT	d-5	72.4±16.7	80.0±27.6	76.5±24.3	78.7±17.5	93.8±29.4
	d21	70.9±25.5	75.7±29.7	77.2±29.0	77.6±25.7	88.5±27.9
	d44	78.0±26.6	82.8±29.9	84.0±34.3	83.7±25.6	93.9±26.1
	d67	83.4±37.3	79.5±8.1	96.3±43.8	104.6±30.9	102.6±43.7
ALP	d-5	391.4±165.0	435.9±186.9	384.3±106.8	403.0±112.3	418.0±102.7
	d21	341.3±161.3	419.3±195.0	349.1±83.8	408.3±174.9	380.6±103.3
	d44	391.4±202.3	453.1±272.9	392.7±153.1	423.3±194.1	403.3±130.8
	d67	358.0±234.7	520.8±372.6	445.5±271.7	548.0±314.7	401.0±186.9
CK	d-5	279.7±106.2	203.8±70.4	211.7±93.3	282.4±174.6	233.7±110.9
	d21	262.9±48.0	199.0±53.1	201.8±46.0	244.2±76.7	219.7±108.6
	d44	245.0±70.8	199.3±34.1	234.0±114.0	217.6±93.2	215.3±103.9
	d67	226.0±61.6	173.8±24.1	165.6±32.9	228.1±80.4	235.7±119.6
TG	d-5	0.46±0.13	0.40±0.17	0.43±0.14	0.41±0.17	0.35±0.11
	d21	0.36±0.13	0.31±0.08	0.36±0.14	0.34±0.13	0.31±0.12
	d44	0.33±0.14	0.26±0.08	0.37±0.12	0.33±0.14	0.27±0.10
	d67	0.34±0.06	0.36±0.07	0.38±0.11	0.49±0.15	0.53±0.21
CHO	d-5	3.06±0.36	3.38±0.63	3.43±0.56	3.35±0.40	3.26±0.57
	d21	3.74±0.54	3.72±0.72	3.85±0.66	3.89±0.83	3.61±0.58
	d44	3.67±0.46	3.76±0.66	3.87±0.57	3.83±0.57	3.71±0.44
	d67	3.48±0.38	3.70±0.45	4.18±0.67	4.20±0.61	4.13±0.70
CREA	d-5	94.0±19.8	89.5±11.5	92.1±16.1	86.2±11.9	93.8±14.1
	d21	87.9±14.4	89.1±13.0	91.8±13.5	94.4±12.9	103.1±16.7
	d44	85.4±16.3	83.2±11.3	91.2±12.0	88.0±12.0	100.6±12.0*
	d67	86.8±7.5	84.6±12.4	105.5±14.0	91.7±13.7	107.8±10.9
BUN	d-5	7.5±0.9	7.8±1.7	7.4±1.2	7.9±1.3	6.1±1.1
	d21	6.7±1.4	7.3±1.9	7.6±1.0	7.7±1.3	7.5±1.4
	d44	6.4±1.1	7.9±2.2	7.3±1.4	7.2±1.7	7.1±1.4
	d67	6.8±0.8	6.0±1.2	6.0±0.8	7.9±1.6	6.4±0.7
TBIL	d-5	5.6±0.6	5.4±0.5	5.4±0.5	5.1±0.5	5.4±0.8
	d21	7.3±1.3	7.2±0.9	7.1±1.8	7.3±1.4	7.5±1.2
	d44	6.1±1.0	6.1±0.7	6.2±0.8	6.4±0.9	6.8±1.0
	d67	6.7±0.9	5.8±0.6	6.9±2.3	6.3±1.1	6.2±1.5
TP	d-5	78.9±3.9	79.1±5.4	80.4±2.9	80.6±4.9	79.9±3.7

Parameter	Time (d)	Vehicle	Low-dose 1.0×10^9 VP/animal	Mid-dose 1.0×10^10 VP/animal	High-dose 1.0×10^11 VP/animal	Ad5-null control 1.0×10^11 VP/animal
	d21	80.3±3.4	81.5±4.3	81.5±3.8	82.7±5.1	85.3±3.6
	d44	77.7±4.2	80.2±4.7	79.4±4.2	79.7±4.8	83.4±3.9
	d67	75.9±4.0	80.8±5.9	85.3±3.1*	81.4±1.5	90.4±3.5*
ALB	d-5	55.0±2.3	56.0±2.7	56.0±1.3	56.0±3.7	56.3±3.0
	d21	55.0±1.8	57.6±1.7	57.8±2.5*	58.4±3.0**	60.9±2.5**
	d44	53.9±1.8	56.3±2.8	55.3±2.6	55.7±2.8	58.7±2.1**
	d67	53.4±2.2	59.4±2.4*	60.5±2.9*	59.1±2.8*	61.3±2.2*
GLU	d-5	5.06±1.04	5.23±1.24	4.97±1.16	5.06±1.08	5.10±1.15
	d21	4.63±0.74	4.38±0.96	4.61±0.80	4.27±0.91	4.23±0.53
	d44	5.11±0.98	4.99±1.21	5.31±0.99	4.80±0.77	4.98±0.41
	d67	5.65±0.58	4.17±0.55	4.88±1.02	4.92±0.17	5.11±0.99
GLO	d-5	23.9±3.3	23.1±4.2	24.4±2.5	24.6±1.8	23.5±4.6
	d21	25.3±2.8	23.9±4.1	23.7±2.9	24.3±3.9	24.4±4.4
	d44	23.8±3.5	24.0±3.8	24.0±3.4	24.0±3.8	24.7±4.0
	d67	22.5±2.9	21.3±5.3	24.8±2.1	22.4±2.9	29.2±4.7
A/G	d-5	2.4±0.3	2.5±0.5	2.3±0.2	2.3±0.1	2.5±0.5
	d21	2.2±0.2	2.5±0.5	2.5±0.4	2.5±0.4	2.6±0.5
	d44	2.3±0.3	2.4±0.5	2.4±0.4	2.4±0.4	2.4±0.4
	d67	2.4±0.3	3.0±1.0	2.5±0.3	2.7±0.5	2.2±0.4

[a]Data expressed as mean ± SD.

[*]$p < 0.05$ compared with the vehicle control.

[**]$p < 0.01$ compared with the vehicle control.
