# Supplementary material for: Immunogenicity of adenovirus-vector vaccine targeting hepatitis B virus: non-clinical safety assessment in non-human primates
Source: Virol J. 2018 Jul 24;15:111. doi: 10.1186/s12985-018-1026-3 (PMC6056916; doi:10.1186/s12985-018-1026-3)
Supplement: Supplementary file 3 — Table S3. Effects of Ad-HBV administration on body temperature. (PDF 173 kb) [file 12985_2018_1026_MOESM3_ESM.pdf]

Additional file 3: Table S3 Effects of Ad-HBV administration on body temperature

Time（d）	N	Vehicle	Low-dose 1.0×10^9 VP/animal	Mid-dose 1.0×10^10 VP/animal	High-dose 1.0×10^11 VP/animal	Ad5-null control 1.0×10^11 VP/animal
d-11	10	39.2±0.5	39.4±0.5	39.1±0.5	39.2±0.4	39.5±0.4
d-3	10	39.0±0.4	39.3±0.5	38.8±0.6	39.0±0.3	39.3±0.4
d5	10	38.8±0.2	39.1±0.4*	39.2±0.2*	39.1±0.3*	39.4±0.3**
d12	10	38.8±0.2	39.0±0.3	39.3±0.4**	39.3±0.2**	39.5±0.4**
d19	10	38.7±0.6	39.3±0.4*	39.2±0.3*	39.3±0.2**	39.5±0.3**
d26	10	38.7±0.2	38.9±0.3	38.9±0.4	39.1±0.2**	39.4±0.4**
d33	10	38.9±0.3	39.2±0.3*	39.2±0.4*	39.3±0.2**	39.6±0.3**
d40	10	38.9±0.3	39.3±0.3**	39.3±0.2*	39.3±0.3*	39.6±0.4**
d45	10	39.0±0.4	39.1±0.4	39.3±0.2	39.2±0.2	39.4±0.4
d54	4	38.8±0.3	39.1±0.4	38.7±0.6	39.0±0.3	39.2±0.3
d61	4	39.3±0.3	39.3±0.4	38.9±0.7	39.5±0.2	38.9±0.2
d67	4	39.1±0.3	39.3±0.5	39.1±0.6	39.2±0.1	39.3±0.3

[a]Expressed as degrees Celsius (mean±SD).

*$p < 0.05$ compared with the vehicle control.

**$p < 0.01$ compared with the vehicle control.
