# Supplementary material for: Immunogenicity of adenovirus-vector vaccine targeting hepatitis B virus: non-clinical safety assessment in non-human primates
Source: Virol J. 2018 Jul 24;15:111. doi: 10.1186/s12985-018-1026-3 (PMC6056916; doi:10.1186/s12985-018-1026-3)
Supplement: Supplementary file 4 — Table S4. Effects of Ad-HBV on Lymphocytes subsets. (DOCX 55 kb) [file 12985_2018_1026_MOESM4_ESM.docx]

**Additional file 4: Table S1 Effects of Ad-HBV administration on Hematology**

| **Parameter** | | **Time**  **(d)** | **Vehicle** | | **Low-dose**  **1.0×10^9 VP/animal** | **Mid-dose**  **1.0×10^10 VP/animal** | **High-dose**  **1.0×10^11 VP/animal** | **Ad5-null control**  **1.0×10^11 VP/animal** |
| --- | --- | --- | --- | --- | --- | --- | --- | --- |
| WBC | | d-5 | 14.08±4.18 | | 12.76±3.33 | 10.78±3.22 | 12.17±2.71 | 13.60±2.30 |
|  |  | d21 | 10.46±2.53 | | 8.81±2.60 | 8.56±2.61 | 10.37±3.14 | 8.16±1.80 |
|  |  | d44 | 12.97±2.66 | | 11.67±3.60 | 10.37±2.75 | 9.55±2.83* | 9.55±2.37* |
|  |  | d67 | 16.39±2.49 | | 12.93±4.86 | 10.91±3.01 | 10.18±2.72 | 11.39±1.54 |
| RBC | | d-5 | 5.25±0.53 | | 5.39±0.56 | 5.30±0.46 | 5.31±0.50 | 5.29±0.27 |
|  |  | d21 | 5.49±0.27 | | 5.39±0.39 | 5.41±0.29 | 5.44±0.35 | 5.39±0.26 |
|  |  | d44 | 5.83±0.33 | | 5.72±0.35 | 5.58±0.37 | 5.62±0.31 | 5.58±0.27 |
|  |  | d67 | 5.71±0.22 | | 5.52±0.19 | 5.61±0.52 | 5.71±0.60 | 5.62±0.49 |
| HGB | | d-5 | 125±12 | | 128±12 | 127±12 | 126±12 | 126±8 |
|  |  | d21 | 133±10 | | 131±8 | 132±7 | 131±8 | 130±5 |
|  |  | d44 | 141±8 | | 138±6 | 136±11 | 134±7 | 135±6 |
|  |  | d67 | 134±4 | | 132±2 | 133±15 | 132±12 | 133±13 |
| HCT | | d-5 | 39.7±3.9 | | 40.7±3.7 | 40.3±3.6 | 40.1±3.4 | 40.2±2.5 |
|  |  | d21 | 42.0±3.1 | | 40.3±2.4 | 40.5±2.0 | 40.6±2.8 | 40.5±1.6 |
|  |  | d44 | 43.6±2.6 | | 42.4±2.0 | 41.7±2.7 | 41.1±2.2 | 41.9±1.7 |
|  |  | d67 | 45.5±3.5 | | 43.7±1.4 | 44.0±5.5 | 44.4±3.3 | 44.5±3.6 |
| MCV | | d-5 | 75.7±4.1 | | 75.7±3.7 | 76.0±2.6 | 75.7±3.0 | 75.9±2.5 |
|  |  | d21 | 76.5±4.4 | | 74.8±2.5 | 75.0±3.3 | 74.7±3.5 | 75.3±2.5 |
|  |  | d44 | 74.9±3.6 | | 74.3±2.4 | 74.8±3.3 | 73.3±3.1 | 75.3±1.9 |
|  |  | d67 | 79.5±4.4 | | 79.2±0.4 | 78.2±4.1 | 78.1±3.9 | 79.4±3.1 |
| MCH | | d-5 | | 23.8±1.3 | 23.9±1.1 | 24.0±1.0 | 23.7±1.2 | 23.8±0.7 |
|  |  | d21 | | 24.3±1.3 | 24.3±1.0 | 24.4±1.0 | 24.1±1.2 | 24.1±0.7 |
|  |  | d44 | | 24.3±1.4 | 24.2±1.0 | 24.3±1.0 | 23.9±1.2 | 24.2±0.8 |
|  |  | d67 | | 23.4±0.7 | 23.9±0.6 | 23.7±0.5 | 23.3±1.8 | 23.7±1.2 |
| MCHC | | d-5 | | 314±8 | 316±10 | 315±11 | 313±14 | 313±7 |
|  |  | d21 | | 318±10 | 325±10 | 325±13 | 322±15 | 320±9 |
|  |  | d44 | | 324±10 | 326±9 | 325±14 | 326±12 | 321±7 |
|  |  | d67 | | 295±16 | 302±7 | 304±14 | 298±14 | 299±11 |
| RDW | | d-5 | | 14.1±1.6 | 13.5±0.8 | 13.7±0.9 | 14.2±0.7 | 14.0±0.9 |
|  |  | d21 | | 13.4±0.8 | 12.8±0.8 | 13.2±0.7 | 13.2±0.7 | 13.3±0.8 |
|  |  | d44 | | 12.2±0.4 | 11.8±0.7 | 12.0±0.5 | 12.1±0.6 | 12.3±0.5 |
|  |  | d67 | | 12.2±0.2 | 11.8±0.5 | 11.9±0.5 | 11.9±0.4 | 12.1±0.4 |
| PLT | | d-5 | | 416±72 | 417±116 | 356±67 | 384±100 | 359±73 |
|  |  | d21 | | 483±72 | 462±129 | 461±105 | 434±98 | 424±95 |
|  |  | d44 | | 428±81 | 405±93 | 400±94 | 371±78 | 359±76 |
|  |  | d67 | | 456±71 | 434±108 | 368±122 | 332±82 | 362±79 |
| MPV | | d-5 | | 9.6±0.8 | 9.1±1.0 | 9.8±1.3 | 9.4±0.9 | 10.0±1.0 |
|  |  | d21 | | 9.1±0.9 | 8.6±0.7 | 9.4±1.1 | 9.3±0.7 | 9.8±1.2 |
|  |  | d44 | | 9.3±0.9 | 9.0±0.7 | 9.5±1.1 | 9.0±0.5 | 9.8±1.4 |
|  |  | d67 | | 9.4±0.9 | 9.0±0.8 | 10.1±1.9 | 9.5±1.0 | 10.0±2.0 |
| PDW | | d-5 | | 46.6±3.3 | 45.3±5.6 | 47.4±6.3 | 48.0±3.9 | 50.6±5.5 |
|  |  | d21 | | 47.8±4.3 | 46.2±3.6 | 47.1±6.6 | 45.5±2.3 | 51.1±4.8 |
|  |  | d44 | | 47.8±5.2 | 47.2±4.3 | 48.8±7.3 | 48.2±3.1 | 53.8±5.2 |
|  |  | d67 | | 48.7±2.7 | 47.6±5.9 | 52.7±9.2 | 49.1±3.9 | 52.6±4.9 |
| PCT | | d-5 | | 0.40±0.05 | 0.38±0.10 | 0.34±0.06 | 0.36±0.08 | 0.36±0.06 |
|  |  | d21 | | 0.43±0.05 | 0.39±0.10 | 0.43±0.08 | 0.40±0.09 | 0.41±0.08 |
|  |  | d44 | | 0.39±0.05 | 0.36±0.07 | 0.37±0.06 | 0.33±0.06 | 0.35±0.06 |
|  |  | d67 | | 0.42±0.04 | 0.39±0.10 | 0.35±0.05 | 0.31±0.05 | 0.35±0.05 |
| %NEUT | | d-5 | | 48.9±9.8 | 44.7±13.6 | 43.2±12.8 | 48.5±16.0 | 50.4±9.9 |
|  |  | d21 | | 36.3±10.4 | 27.6±8.0 | 31.9±10.1 | 45.0±15.6 | 35.1±6.8 |
|  |  | d44 | | 34.2±5.6 | 33.0±9.8 | 37.4±13.8 | 36.5±14.4 | 39.8±10.4 |
|  |  | d67 | | 43.2±10.1 | 30.8±10.6 | 31.3±9.7 | 33.3±14.3 | 38.9±10.3 |
| %LYMPH | | d-5 | | 45.7±9.4 | 49.9±13.2 | 45.3±12.5 | 45.4±15.4 | 44.8±9.3 |
|  |  | d21 | | 56.8±9.8 | 65.5±8.7 | 60.5±8.6 | 48.9±14.5 | 59.2±6.5 |
|  |  | d44 | | 58.6±5.0 | 61.1±10.0 | 55.5±12.5 | 55.3±13.4 | 54.9±9.5 |
|  |  | d67 | | 50.2±9.0 | 64.9±10.6 | 61.9±8.7 | 61.4±14.5 | 56.5±10.2 |
| %MONO | | d-5 | | 3.1±0.5 | 3.1±0.5 | 9.6±16.1 | 3.9±1.5 | 3.0±0.8 |
|  |  | d21 | | 4.0±1.3 | 3.3±0.9 | 4.6±1.3 | 3.7±1.5 | 3.2±1.0 |
|  |  | d44 | | 3.8±0.9 | 3.2±0.7 | 4.9±1.3 | 5.7±2.3 | 3.7±1.1 |
|  |  | d67 | | 3.5±0.2 | 2.6±0.3 | 4.4±1.0 | 3.3±1.3 | 3.2±1.4 |
| %EOS | | d-5 | | 1.5±1.7 | 1.7±1.6 | 1.3±1.1 | 1.5±1.8 | 1.2±0.7 |
|  |  | d21 | | 2.1±1.6 | 2.7±2.0 | 2.3±2.4 | 1.7±1.2 | 1.6±1.2 |
|  |  | d44 | | 2.6±2.7 | 1.9±1.3 | 1.6±1.7 | 1.8±2.0 | 0.9±0.5 |
|  |  | d67 | | 2.4±2.1 | 1.0±0.6 | 1.8±1.2 | 1.5±0.9 | 0.8±0.7 |
| %BASO | | d-5 | | 0.2±0.1 | 0.2±0.0 | 0.2±0.1 | 0.2±0.1 | 0.2±0.1 |
|  |  | d21 | | 0.2±0.1 | 0.3±0.1 | 0.2±0.1 | 0.2±0.1 | 0.3±0.1 |
|  |  | d44 | | 0.2±0.1 | 0.2±0.1 | 0.2±0.1 | 0.2±0.1 | 0.2±0.1 |
|  |  | d67 | | 0.3±0.1 | 0.3±0.1 | 0.3±0.1 | 0.2±0.1 | 0.2±0.1 |
| %LUC | | d-5 | | 0.6±0.2 | 0.5±0.2 | 0.4±0.1 | 0.5±0.2 | 0.5±0.2 |
|  |  | d21 | | 0.6±0.2 | 0.6±0.1 | 0.5±0.2 | 0.5±0.2 | 0.7±0.2 |
|  |  | d44 | | 0.6±0.2 | 0.5±0.1 | 0.4±0.2 | 0.5±0.2 | 0.5±0.2 |
|  |  | d67 | | 0.6±0.2 | 0.5±0.1 | 0.4±0.2 | 0.4±0.1 | 0.5±0.2 |
| %RETIC | d-5 | | 2.32±1.43 | | 2.07±0.47 | 2.09±0.64 | 2.24±0.71 | 2.01±0.46 |
|  | d21 | | 1.77±0.52 | | 1.71±0.56 | 1.77±0.52 | 1.48±0.42 | 1.68±0.22 |
|  | d44 | | 1.02±0.33 | | 1.14±0.31 | 1.22±0.42 | 1.03±0.36 | 1.19±0.37 |
|  | d67 | | 1.22±0.11 | | 0.90±0.16 | 1.13±0.11 | 0.82±0.16 | 1.14±0.42 |

^a^Data expressed as mean±SD.

^*^*p＜0.05* compared with the vehicle control.

**Additional table S2. Effects of Ad-HBV administration on serum chemistry**

| **Parameter** | **Time**  **(d)** | **Vehicle** | **Low-dose**  **1.0×10^9 VP/animal** | **Mid-dose**  **1.0×10^10 VP/animal** | **High-dose**  **1.0×10^11 VP/animal** | **Ad5-null control**  **1.0×10^11 VP/animal** |
| --- | --- | --- | --- | --- | --- | --- |
| ALT | d-5 | 92.6±57.6 | 77.3±39.7 | 82.9±61.3 | 72.7±23.8 | 90.6±47.5 |
|  | d21 | 54.1±13.2 | 59.1±31.5 | 54.4±17.8 | 76.5±47.1 | 87.2±50.0 |
|  | d44 | 70.1±28.6 | 77.9±42.0 | 60.4±21.3 | 82.1±58.6 | 98.2±53.8 |
|  | d67 | 57.8±11.1 | 54.0±23.3 | 77.3±69.5 | 84.5±65.8 | 162.5±140.5 |
| AST | d-5 | 63.2±21.0 | 66.8±24.8 | 60.3±15.7 | 60.5±10.0 | 54.8±11.7 |
|  | d21 | 51.1±7.8 | 63.1±18.7 | 52.6±13.9 | 53.9±13.9 | 49.1±11.1 |
|  | d44 | 44.3±10.4 | 53.0±17.5 | 43.7±7.6 | 45.1±13.4 | 39.8±7.6 |
|  | d67 | 37.7±2.4 | 47.4±15.5 | 33.4±11.5 | 45.0±13.3 | 43.0±14.6 |
| GGT | d-5 | 72.4±16.7 | 80.0±27.6 | 76.5±24.3 | 78.7±17.5 | 93.8±29.4 |
|  | d21 | 70.9±25.5 | 75.7±29.7 | 77.2±29.0 | 77.6±25.7 | 88.5±27.9 |
|  | d44 | 78.0±26.6 | 82.8±29.9 | 84.0±34.3 | 83.7±25.6 | 93.9±26.1 |
|  | d67 | 83.4±37.3 | 79.5±8.1 | 96.3±43.8 | 104.6±30.9 | 102.6±43.7 |
| ALP | d-5 | 391.4±165.0 | 435.9±186.9 | 384.3±106.8 | 403.0±112.3 | 418.0±102.7 |
|  | d21 | 341.3±161.3 | 419.3±195.0 | 349.1±83.8 | 408.3±174.9 | 380.6±103.3 |
|  | d44 | 391.4±202.3 | 453.1±272.9 | 392.7±153.1 | 423.3±194.1 | 403.3±130.8 |
|  | d67 | 358.0±234.7 | 520.8±372.6 | 445.5±271.7 | 548.0±314.7 | 401.0±186.9 |
| CK | d-5 | 279.7±106.2 | 203.8±70.4 | 211.7±93.3 | 282.4±174.6 | 233.7±110.9 |
|  | d21 | 262.9±48.0 | 199.0±53.1 | 201.8±46.0 | 244.2±76.7 | 219.7±108.6 |
|  | d44 | 245.0±70.8 | 199.3±34.1 | 234.0±114.0 | 217.6±93.2 | 215.3±103.9 |
|  | d67 | 226.0±61.6 | 173.8±24.1 | 165.6±32.9 | 228.1±80.4 | 235.7±119.6 |
| TG | d-5 | 0.46±0.13 | 0.40±0.17 | 0.43±0.14 | 0.41±0.17 | 0.35±0.11 |
|  | d21 | 0.36±0.13 | 0.31±0.08 | 0.36±0.14 | 0.34±0.13 | 0.31±0.12 |
|  | d44 | 0.33±0.14 | 0.26±0.08 | 0.37±0.12 | 0.33±0.14 | 0.27±0.10 |
|  | d67 | 0.34±0.06 | 0.36±0.07 | 0.38±0.11 | 0.49±0.15 | 0.53±0.21 |
| CHO | d-5 | 3.06±0.36 | 3.38±0.63 | 3.43±0.56 | 3.35±0.40 | 3.26±0.57 |
|  | d21 | 3.74±0.54 | 3.72±0.72 | 3.85±0.66 | 3.89±0.83 | 3.61±0.58 |
|  | d44 | 3.67±0.46 | 3.76±0.66 | 3.87±0.57 | 3.83±0.57 | 3.71±0.44 |
|  | d67 | 3.48±0.38 | 3.70±0.45 | 4.18±0.67 | 4.20±0.61 | 4.13±0.70 |
| CREA | d-5 | 94.0±19.8 | 89.5±11.5 | 92.1±16.1 | 86.2±11.9 | 93.8±14.1 |
|  | d21 | 87.9±14.4 | 89.1±13.0 | 91.8±13.5 | 94.4±12.9 | 103.1±16.7 |
|  | d44 | 85.4±16.3 | 83.2±11.3 | 91.2±12.0 | 88.0±12.0 | 100.6±12.0* |
|  | d67 | 86.8±7.5 | 84.6±12.4 | 105.5±14.0 | 91.7±13.7 | 107.8±10.9 |
| BUN | d-5 | 7.5±0.9 | 7.8±1.7 | 7.4±1.2 | 7.9±1.3 | 6.1±1.1 |
|  | d21 | 6.7±1.4 | 7.3±1.9 | 7.6±1.0 | 7.7±1.3 | 7.5±1.4 |
|  | d44 | 6.4±1.1 | 7.9±2.2 | 7.3±1.4 | 7.2±1.7 | 7.1±1.4 |
|  | d67 | 6.8±0.8 | 6.0±1.2 | 6.0±0.8 | 7.9±1.6 | 6.4±0.7 |
| TBIL | d-5 | 5.6±0.6 | 5.4±0.5 | 5.4±0.5 | 5.1±0.5 | 5.4±0.8 |
|  | d21 | 7.3±1.3 | 7.2±0.9 | 7.1±1.8 | 7.3±1.4 | 7.5±1.2 |
|  | d44 | 6.1±1.0 | 6.1±0.7 | 6.2±0.8 | 6.4±0.9 | 6.8±1.0 |
|  | d67 | 6.7±0.9 | 5.8±0.6 | 6.9±2.3 | 6.3±1.1 | 6.2±1.5 |
| TP | d-5 | 78.9±3.9 | 79.1±5.4 | 80.4±2.9 | 80.6±4.9 | 79.9±3.7 |
|  | d21 | 80.3±3.4 | 81.5±4.3 | 81.5±3.8 | 82.7±5.1 | 85.3±3.6 |
|  | d44 | 77.7±4.2 | 80.2±4.7 | 79.4±4.2 | 79.7±4.8 | 83.4±3.9 |
|  | d67 | 75.9±4.0 | 80.8±5.9 | 85.3±3.1* | 81.4±1.5 | 90.4±3.5* |
| ALB | d-5 | 55.0±2.3 | 56.0±2.7 | 56.0±1.3 | 56.0±3.7 | 56.3±3.0 |
|  | d21 | 55.0±1.8 | 57.6±1.7 | 57.8±2.5* | 58.4±3.0** | 60.9±2.5** |
|  | d44 | 53.9±1.8 | 56.3±2.8 | 55.3±2.6 | 55.7±2.8 | 58.7±2.1** |
|  | d67 | 53.4±2.2 | 59.4±2.4* | 60.5±2.9* | 59.1±2.8* | 61.3±2.2* |
| GLU | d-5 | 5.06±1.04 | 5.23±1.24 | 4.97±1.16 | 5.06±1.08 | 5.10±1.15 |
|  | d21 | 4.63±0.74 | 4.38±0.96 | 4.61±0.80 | 4.27±0.91 | 4.23±0.53 |
|  | d44 | 5.11±0.98 | 4.99±1.21 | 5.31±0.99 | 4.80±0.77 | 4.98±0.41 |
|  | d67 | 5.65±0.58 | 4.17±0.55 | 4.88±1.02 | 4.92±0.17 | 5.11±0.99 |
| GLO | d-5 | 23.9±3.3 | 23.1±4.2 | 24.4±2.5 | 24.6±1.8 | 23.5±4.6 |
|  | d21 | 25.3±2.8 | 23.9±4.1 | 23.7±2.9 | 24.3±3.9 | 24.4±4.4 |
|  | d44 | 23.8±3.5 | 24.0±3.8 | 24.0±3.4 | 24.0±3.8 | 24.7±4.0 |
|  | d67 | 22.5±2.9 | 21.3±5.3 | 24.8±2.1 | 22.4±2.9 | 29.2±4.7 |
| A/G | d-5 | 2.4±0.3 | 2.5±0.5 | 2.3±0.2 | 2.3±0.1 | 2.5±0.5 |
|  | d21 | 2.2±0.2 | 2.5±0.5 | 2.5±0.4 | 2.5±0.4 | 2.6±0.5 |
|  | d44 | 2.3±0.3 | 2.4±0.5 | 2.4±0.4 | 2.4±0.4 | 2.4±0.4 |
|  | d67 | 2.4±0.3 | 3.0±1.0 | 2.5±0.3 | 2.7±0.5 | 2.2±0.4 |

^a^Data expressed as mean±SD.

^*^*p＜0.05* compared with the vehicle control.

^**^*p＜0.01* compared with the vehicle control.

**Additional table S3. Effects of Ad-HBV administration on body temperature**

| **Time**  **（d）** | **N** | **Vehicle** | **Low-dose**  **1.0×10^9 VP/animal** | **Mid-dose**  **1.0×10^10 VP/animal** | **High-dose**  **1.0×10^11 VP/animal** | **Ad5-null control**  **1.0×10^11 VP/animal** |
| --- | --- | --- | --- | --- | --- | --- |
| d-11 | 10 | 39.2±0.5 | 39.4±0.5 | 39.1±0.5 | 39.2±0.4 | 39.5±0.4 |
| d-3 | 10 | 39.0±0.4 | 39.3±0.5 | 38.8±0.6 | 39.0±0.3 | 39.3±0.4 |
| d5 | 10 | 38.8±0.2 | 39.1±0.4* | 39.2±0.2* | 39.1±0.3* | 39.4±0.3** |
| d12 | 10 | 38.8±0.2 | 39.0±0.3 | 39.3±0.4** | 39.3±0.2** | 39.5±0.4** |
| d19 | 10 | 38.7±0.6 | 39.3±0.4* | 39.2±0.3* | 39.3±0.2** | 39.5±0.3** |
| d26 | 10 | 38.7±0.2 | 38.9±0.3 | 38.9±0.4 | 39.1±0.2** | 39.4±0.4** |
| d33 | 10 | 38.9±0.3 | 39.2±0.3* | 39.2±0.4* | 39.3±0.2** | 39.6±0.3** |
| d40 | 10 | 38.9±0.3 | 39.3±0.3** | 39.3±0.2* | 39.3±0.3* | 39.6±0.4** |
| d45 | 10 | 39.0±0.4 | 39.1±0.4 | 39.3±0.2 | 39.2±0.2 | 39.4±0.4 |
| d54 | 4 | 38.8±0.3 | 39.1±0.4 | 38.7±0.6 | 39.0±0.3 | 39.2±0.3 |
| d61 | 4 | 39.3±0.3 | 39.3±0.4 | 38.9±0.7 | 39.5±0.2 | 38.9±0.2 |
| d67 | 4 | 39.1±0.3 | 39.3±0.5 | 39.1±0.6 | 39.2±0.1 | 39.3±0.3 |

^a^Expressed as degrees Celsius (mean±SD).

**p＜0.05* compared with the vehicle control.

***p＜0.01* compared with the vehicle control.

**Additional table S4. Effects of Ad-HBV on Lymphocytes subsets**

| **Parameter** | **Time**  **(d)** | **Vehicle** | **Low-dose**  **1.0×10^9 VP/animal** | **Mid-dose**  **1.0×10^10 VP/animal** | **High-dose**  **1.0×10^11 VP/animal** | **Ad5-null control**  **1.0×10^11 VP/animal** |
| --- | --- | --- | --- | --- | --- | --- |
| CD3+CD8+  T cell | d-5 | 11.8±3.9 | 15.1±5.6 | 13.7±4.8 | 17.8±8.5 | 11.4±6.3 |
|  | d3 | 22.2±5.3 | 26.5±7.2 | 19.8±4.5 | 20.2±6.2 | 23.9±3.9 |
|  | d10 | 14.9±6.6 | 19.6±5.1 | 18.3±5.2 | 21.9±7.1 | 19.0±3.5 |
|  | d45 | 9.7±2.6 | 14.2±3.6** | 10.8±3.4 | 11.8±3.5 | 10.0±2.2 |
|  | d68 | 21.7±6.2 | 21.5±5.7 | 22.4±6.0 | 22.9±5.4 | 24.6±5.4 |
| CD3+CD4+  T cell | d-5 | 21.7±8.1 | 21.3±6.9 | 24.3±9.1 | 19.6±4.5 | 19.7±8.6 |
|  | d3 | 22.0±4.4 | 24.9±3.3 | 26.8±5.1 | 23.5±4.1 | 23.0±5.2 |
|  | d10 | 28.8±11.3 | 32.8±6.8 | 31.6±7.0 | 31.3±5.7 | 33.0±3.7 |
|  | d45 | 12.6±5.6 | 15.6±5.0 | 12.7±4.4 | 13.2±4.0 | 13.2±2.5 |
|  | d68 | 18.7±6.8 | 16.9±5.5 | 20.8±4.0 | 19.0±6.7 | 18.8±6.9 |
|  | d68 | 0.8±0.1 | 0.8±0.1 | 1.0±0.2 | 0.9±0.5 | 0.8±0.5 |
| CD45+CD14+  macrophage | d-5 | 1.2±1.2 | 0.8±0.7 | 1.2±0.7 | 0.7±0.4 | 0.6±0.4 |
|  | d3 | 1.0±0.8 | 0.7±0.5 | 1.0±1.0 | 0.6±0.7 | 0.6±0.7 |
|  | d10 | 1.9±0.8 | 2.5±0.9 | 2.5±0.8 | 1.6±0.7 | 1.5±0.6 |
|  | d45 | 0.7±0.4 | 1.2±0.8 | 1.2±0.5 | 1.1±0.5 | 1.6±0.5** |
|  | d68 | 1.4±0.7 | 1.0±0.8 | 1.6±0.6 | 1.4±1.0 | 1.4±1.0 |

^a^Data expressed as mean±SD.

^*^*p＜0.05* compared with the vehicle control.

^**^*p＜0.01* compared with the vehicle control.
